# Relationship between Anthropometric Measures and Anxiety Perception in Soccer Players

**DOI:** 10.3390/ijerph19158898

**Published:** 2022-07-22

**Authors:** Alejandro Martínez-Rodríguez, Marcelo Peñaranda-Moraga, Manuel Vicente-Martínez, Miguel Martínez-Moreno, Bernardo J. Cuestas-Calero, Jorge Soler-Durá, Rodrigo Yáñez-Sepúlveda, Antonio Jesús Muñoz-Villena

**Affiliations:** 1 Department of Analytical Chemistry, Nutrition and Food Science, Alicante Institute for Health and Biomedical Research (ISABIAL), University of Alicante, 03690 Alicante, Spain; 2 European Institute of Exercise and Health (EIEH), University of Alicante, 03690 Alicante, Spain; marcelopenaranda.eieh@gmail.com (M.P.-M.); miguelmartinez.eieh@gmail.com (M.M.-M.); jorgesoler.eieh@gmail.com (J.S.-D.); 3 Faculty of Health Science, Miguel de Cervantes European University, 47012 Valladolid, Spain; mvmartinez11006@alumnos.uemc.es; 4 Faculty of Sport, Catholic University San Antonio of Murcia, 30107 Murcia, Spain; bjcuestas@alu.ucam.edu; 5 Escuela de Educación, Pedagogía en Educación Física, Universidad Viña del Mar, Viña del Mar 7055, Chile; rodrigo.yanez@uvm.cl; 6 Department of Social Psychology and Communication, University of Alicante, 03690 Alicante, Spain

**Keywords:** anthropometry, sports performance, health, body composition, maturation, psychology, anxiety, soccer

## Abstract

In the sports context, it has been corroborated that the physical demands of presenting an “ideal” body configuration have been associated with different psychological variables, such as self-esteem, anxiety and personality dimensions, such as perfectionism. Specifically, there is evidence that anthropometric measures may be closely related to psychological indicators. A total of 33 male soccer players (18.12 ± 1.24 years) participated in the investigation. Anthropometric assessments were carried out following the ISAK standards for the restricted profile. All of them completed the Competitive Trait Anxiety Inventory (CTAI-2D) in its Spanish version. The percent fat was calculated using Withers (density) and Siri equations. The ∑7 skinfolds were used to calculate this. After statistical analysis, significant mean differences were observed in the somatic anxiety dimension (valence) and a medium–large effect size. Regarding correlations, the significantly negative relationship between self-confidence (intentionality) and somatic anxiety (valence) was noteworthy. The relationship between psychological variables and anthropometric measurements was corroborated, showing the need for interdisciplinary work between psychologists and nutritionists who do not ignore the physical health and psychological well-being of the soccer player.

## 1. Introduction

Soccer is one of the most popular and best known sports around the world [1], being of interest for researchers to study the different determinants of sports performance in elite soccer players.

It is possible to attribute successful performances to the athlete’s physical abilities; however, the complexity of the sporting reality evidences other abilities than body and talent. There is evidence that, in addition to physique, game intelligence, psychological skills, athletic characteristics, individual differences, general physical condition and body composition represent essential characteristics in the composition of a soccer player.

Considering that soccer is characterized as an intermittent activity, with events changing every 3 to 5 s, it is well understood that players must be prepared for intense actions that include jumping, turning, tackling, high-speed running and sprinting [2,3]. In addition to the above, the social magnitude of soccer, together with a demanding and uncertain sporting and extra-sporting context, exposes the player to situations that he may perceive as a threat, perceiving depending on the individual anxiogenic characteristics (intensity), interpreting them as facilitators or enhancers of sporting performance (courage/direction).

From a multidimensional perspective, competitive anxiety is defined as the emotional mechanism from which the soccer player responds through his coping styles to situations of threat and uncertainty and which has three components: cognitive, somatic and behavioral. Likewise, anxiety expresses the negative expectations and concerns about oneself, the situation and the possible consequences (cognitive anxiety); it includes affective and physiological elements of the experience derived from autonomic activation (somatic anxiety); and it implies the degree of certainty that the soccer player has about his capacity to succeed in the next competition (self-confidence).

However, depending on its manifestation in behavior or not, it is necessary to distinguish trait anxiety and state anxiety [4]. The trait refers to individual, stable and dispositional differences, while the state is characterized by its modifiability over time. In addition, trait anxiety varies little in the individual, interacting and modifying state anxiety, i.e., athletes with elevated trait anxiety tendencies are more likely to perceive situations as threatening and present a vulnerability to experiencing state anxiety [4,5,6,7].

Therefore, the media impact together with a demanding/pressure sports context that also demands results with immediacy impose an ideal body composition through a weight that assures performance; supposing, on occasions, a threat depending on the interpretation of the player and the consequences derived from his behavior. It has been corroborated that the physical demands for presenting an “ideal” body configuration have been associated with different psychological variables, such as self-esteem [8], anxiety [9] and personality dimensions, such as perfectionism [10].

Body composition involves the analysis of the human body based on the fractionation of total body mass. In the field of sport, its evaluation is important because body composition is among the factors that can determine athletic potential and the probability of success in a sport in combination with technical-tactical, physical, functional and psychosocial factors [11].

In the case of soccer, body fat must be controlled, as adequate levels of fat allow players to move more efficiently during training and matches. Excess adipose tissue will burden the player with useless extra weight [12,13].

Previous research has shown positive correlations between fat mass (%) and time spent during sprint and change of direction tests [14]. It has also been observed to have a negative impact on aerobic and anaerobic capacity, strength, power and speed [3,15].

Therefore, body composition is closely related to the ability of players to achieve maximum performance in all their actions in the game, while teams with higher physical condition and low-fat percentages play in the best leagues and championships [16]. Success and a high level of sportsmanship consequently generate self-confidence [17]. Physical self-perception is the main characteristic of the search for mental health and well-being.

However, it should also be noted that athletes who may experience pressure to lose weight or fat mass may experience low energy availability (LEA) due to disordered eating behavior, inadvertently due to lack of appetite or poor nutritional knowledge, or intentionally to achieve a discipline-specific physique to optimize performance [18]. LEA can result in adverse health outcomes, increased risk of musculoskeletal injury and decreased athletic performance. In addition, male athletes appear to be at risk of lower testosterone levels and associated symptoms of hypogonadal status [18].

Different approaches can be used to evaluate and determine body composition. Among them is the four-component fractionation method, which divides the human body into adipose mass, muscle mass, bone mass and residual mass, based on anthropometric measurements of body mass, skinfolds, perimeters and body diameters [19,20]. There are different equations to determine the percentage of body fat; however, previous research [21] points out that there are no significant differences between the equations of three or seven skinfolds.

In this context, the main aim of this research was to analyze body composition (anthropometrics) and psychological aspects of male soccer players. As initial hypotheses, we planned: Hypothesis 1 (H1). Soccer players with lower percentage of fat mass will show higher levels of self-confidence and lower levels of somatic and cognitive anxiety intensity; Hypothesis 2 (H2). Soccer players with lower percent fat mass will show higher levels of self-confidence and lower levels of valence/direction for sport performance of somatic and cognitive anxiety; Hypothesis 3 (H3). Players who interpret cognitive anxiety as hindering sport performance will exhibit higher levels of fat mass and folds.

## 2. Materials and Methods

### 2.1. Study Design

A descriptive, cross-sectional study was used to analyze body composition through anthropometric measures, anxiety intensity and directionality in male soccer players. The research was conducted in accordance with the ethical standards recognized by the Declaration of Helsinki. The study was approved by the Ethical Committee of the University of Alicante (UA-2021-03-11).

### 2.2. Subjects

The study sample consisted of 33 male soccer players. The participants were recruited from an Academy of Laliga team (Spain). They were under 18 (17.12 ± 1.24 years) professional soccer players with more than 10 years of training experience. Goalkeepers were not included. The measurements were performed during a competitive period. All players received information on the research objectives, experimental protocol and study procedures. Each participant signed the informed consent document. In the case of underage players, parents or legal guardians gave their permission. The anonymity of all participants was preserved.

### 2.3. Anthropometric Data

The anthropometric variables of each subject were measured. For this purpose, a complete profile was drawn up, following the standard protocol of the International Society for the Advancement of Kinanthropometry (ISAK) [22].

All measurements were performed by the same investigator, an ISAK level 2 anthropometrist. The mean technical error was less than 1% for the perimeters, circumferences, lengths and heights, and less than 5% for the skinfolds. All measurements were performed under baseline conditions, in the same location and at room temperature (22 ± 1 °C).

The following approved and previously calibrated anthropometric material was used to perform the measurements: wall-mounted measuring rod (accuracy, 1 mm); digital scale (BC545N, Tanita, Tokyo, Japan; accuracy, 100 g); narrow, inextensible metal tape measure (Lufkin, TX, USA; accuracy, 1 mm); small sliding caliper (Holtain, Crymych, UK; accuracy, 1 mm); skinfold caliper (Harpenden, UK; accuracy, 1 mm); complementary material (demographic pencil to mark the players) and anthropometric bench of 40 × 50 × 30 cm.

Body density was calculated using Withers equation, 1987 [23], using four skinfolds. Then, body fat was determined by Siri equation [24] for the densities previously calculated. The ∑7 skinfolds were calculated using triceps, subscapular, biceps, supraspinal, abdominal, thigh and leg skinfolds (in mm).

### 2.4. Competitive Trait Anxiety Inventory, (CTAI-2D)

Adapted for Spanish athletes by Muñoz-Villena et al. [25]. This instrument is composed of 27 items that evaluate three factors: cognitive anxiety (“I am worried about not doing as well as I could”), somatic anxiety (“I feel nervous before the competition”) and self-confidence (“I usually feel relaxed before competing”); and two scales: intensity and valence/direction. On the one hand, the intensity scale is answered in four response alternatives presented on a Likert-type scale of 4 categories, where 1 corresponds to “not at all”, and 4 corresponds to “very much”. On the other hand, in the category of valence/direction, the alternatives of response vary from “−3: very negative” to “+3: very positive”. The overall reliability index of this research was 0.78.

### 2.5. Statistical Analysis

Statistical analysis of the data was carried out using Jamovi 1.1.3.0. To show the characteristics of the participants, descriptive statistics were performed for all variables (Mean ± SD). Shapiro–Wilk and Levene tests were applied to check the normality of the sample. Analysis of covariance (ANCOVA) with the correction of Tukey’s was used to compare the differences between groups of fat percentage controlling the effect of BMI. Statistical significance was set at p < 0.05. Cohen’s d was used as a measure of the effect size (ES) of the differences between both groups, which were divided according to the percentage of fat mass (<8% or ≥8% calculated with the Withers and Siri equations). This cut-off point was used because previous research [21] conducted in professional soccer players estimated that the mean % fat, without taking goalkeepers into account, was 7.8 ± 1.5%. The correlations were determined using Pearson’s correlation coefficient, with 95% confidence intervals (CI).

## 3. Results

A total of 33 soccer players participated in the study. The global anthropometric characteristics of the participants are presented in Table 1. The players were divided into two groups, according to their % fat mass. The <8% group consisted of a total of 20 players, while the ≥8 group consisted of 13 players.

Table 2 shows the results of the anxiety dimensions (intensity and valence/direction) as a function of fat mass percentage. Significant mean differences were observed in the somatic anxiety dimension (valence) and a medium–large effect size. Specifically, higher mean scores in somatic anxiety (valence) were found in soccer players with a higher fat percentage compared to those with a lower fat mass score.

On the other hand, in the correlational analysis (Table 3), positive associations were observed between somatic anxiety (intensity) and somatic anxiety (direction); likewise, a positive relationship was found between self-confidence (intensity) and self-confidence (direction). On the contrary, there was a significantly negative relationship between self-confidence (intentionality) and somatic anxiety (valence). An inverse relationship was found between somatic anxiety (valence/direction) and the variables: 7-fold sum and fat mass, the latter two variables being positively associated.

## 4. Discussion

In recent decades, researchers have coincided in highlighting the importance of adding other variables (body composition, tactical intelligence, psychological skills) to the physical characteristics of the soccer player for the study of sports performance. There are few studies that unify the study of anthropometric and psychological variables in the sports context. Specifically, the main objective of the present study was to examine the percentage of fat and skinfolds in professional soccer players and the relationship with the intensity and directionality of anxiety.

Comparing the results obtained with previous research, it is observed that the values of the studied athletes are slightly higher, both for somatic and cognitive anxiety, as well as for self-confidence, both with respect to the averages of the first and second teams of the Croatian league [26].

Regarding the first initial hypothesis of this research, in relation to the comparison of soccer players based on the level of fat and the components of anxiety, it is partially fulfilled, since differences were found, although they were not significant. Thus, from previous research with other populations [27], it was expected that athletes with a higher fat mass index would show lower mean scores in the levels of self-confidence. This difference would explain how soccer players with a low level of fat mass would have greater conviction to achieve the goal and succeed in their sporting performance, i.e., greater self-confidence. This could be due to the important role played in performance by body composition in soccer [12,13,28,29], together with the soccer player’s efficacy beliefs through the influence on motivation and cognitive processes, as has been demonstrated in athletes of other sports [30]. Soccer players would increase their confidence level based on a “regulatory mechanism”, which would include physical/mental preparation and body composition.

Regarding the second hypothesis, significant differences were found concerning the differential analysis of the fat mass index in the dimension of somatic anxiety (valence); therefore, the results confirm the hypothesis raised. In this comparison, those soccer players who show a higher percentage of fat present a more negative score in the interpretation of the interference of somatic anxiety in sports performance. Players with higher fat mass scores present a greater negative emotional state, including feelings of nervousness, worry and apprehension related to organismic arousal. This finding suggests that players with a higher fat component are more insecure about their physical characteristics; therefore, if there is an injury, the recovery presents a greater difficulty and duration due to the lack of physical condition. As seen previously [26], the best way to increase sporting success is to accept the fact that anxiety exists but also to keep it at an optimal level. The optimal state of anxiety best suited for sporting success is not too high but not too low either.

Finally, regarding the third hypothesis, those soccer players who interpret somatic anxiety as an enhancer of sports performance present lower scores in the folds measure. This finding is in line with initial studies that have related personality dimensions, such as neuroticism and perfectionism, to skinfolds [10]. This relationship would explain that those soccer players with neuroticism tendencies or who are dissatisfied with their body image are more anxious, influencing the mechanisms of food choice and intake, and hindering their adherence to an adequate and individualized nutritional dietary program. In this sense, previous research indicates that the perception of directionality is more sensitive in distinguishing individual differences, i.e., how the athlete interprets the enhancing or debilitating effects of anxiety on performance [4,6,7,31,32,33]. Identifying the state of body image anxiety of a person at a given time is the starting point for establishing useful and assertive care strategies to help improve body image, for which it is essential to have tools to measure the level and magnitude of this anxiety [34].

As has been observed in the present study, the athletes have low levels of fat mass. This fact leads to the fact that there is no psychological alteration (no significant differences in intensity) that produces an increase in the levels of anxiety in these groups. Thus, it should be emphasized and even presented in a transfer to public health that low levels of fat mass (promoted by healthy and adequate eating habits, together with regular sports practice) have a positive effect on health at the psychological level.

The main limitation is the size of the sample, which limits the generalization of the results obtained to the population and context studied. In addition, the sample is only composed of men. A greater temporal follow-up of the body measurements in the sample under study through longitudinal studies would be desirable, with which to observe if there are variations within the competitive period. The second limitation comes from the measurement of psychological variables, since it is based on a self-report instrument; therefore, it may contain biases derived from social desirability. As for the method of measuring body composition, anthropometry was used and not DXA, which is considered the gold standard for body composition assessments. In addition to the above, it is appropriate to include other variables, such as personality or perception and satisfaction with the athlete’s body image because of their relationship with the development of eating disorders [35] and mental health [34,36], as well as for the possibility of providing greater information of the relationship between psychological constructs and anthropometric measures.

In the same way, it would be useful to carry out more complex statistical techniques that make it possible to establish predictive models, as has been done in other sports contexts [30,37].

Based on the results found, in future research, it would be necessary to corroborate this association between variables with a female sample, as well as to analyze other high-performance sports contexts than soccer. However, this study is an initial approximation, carried out in combination by professionals from the applied and scientific fields, where, according to the ease of access to the sample, they could approach the subject under analysis. Despite the suggested future proposals, the study of anthropometric measures and their relationship with the directionality attributed by the athlete to the anxiety response opens a line of research of interest to continue providing a greater understanding of the reality of the soccer player and the agents that surround him/her, through the identification of variables related to sports performance.

## 5. Conclusions

The research was carried out with the main objective of determining the relationships between body composition (anthropometry) and psychological aspects of male professional soccer players. The conclusion, based on the results of this research, is that there were no significant differences in the intensity of anxiety components in soccer players with low fat levels. The results of somatic anxiety directionality did differ significantly in soccer players of higher or lower than 8% fat; however this did not occur with cognitive anxiety and self-confidence. Greater negative directionality of somatic anxiety translates into sports performance.

## Figures and Tables

**Table 1:** Descriptive statistics of body composition of the total sample.

	Mean	SD
Age (years)	17.12	1.24
Body Height (cm)	179	7.10
Body Weight (kg)	69.1	13.3
BMI	22.0	1.79
∑7 skinfolds	48.1	8.22
Body Fat (%)	7.84	1.30

Data are shown as mean and standard deviation. cm = centimeters; kg = kilograms; BMI = body mass index; % = percentage; ∑ = summation; SD = standard deviation. Body fat (%) equation was Withers-Siri.

**Table 2:** Difference of means in anxiety dimensions as a function of fat mass percentage.

	<8% Fat Mass		≥8% Fat Mass			
	Mean	SD	Mean	SD	*p*	ES
Somatic anxiety (intensity)	17.3	2.75	16.5	2.50	0.383	0.315
Cognitive anxiety (intensity)	24.3	4.21	25.1	3.17	0.574	−0.202
Self-confidence (intensity)	29.2	4.01	28.2	3.24	0.471	0.259
Somatic anxiety (valence)	−3.90	6.25	−8.54	5.80	0.040 *	0.763
Cognitive anxiety (valence)	1.30	9.39	4.25	7.86	0.369	−0.333
Self-confidence (valence)	17.6	7.40	17.2	6.81	0.886	0.051

Data are shown as mean, standard deviation. The *p* and ES values of the analysis of covariance (ANCOVA), using BMI as a covariate, are shown. % = percentage; SD = standard deviation; * *p* < 0.05.

**Table 3:** Correlations between anxiety dimensions, age and anthropometric measures.

	Age (Years)	SA (I)	CA (I)	SC (I)	SA (D)	CA (D)	SC (D)	BW (kg)	∑7 Skinfolds	BF (%)
Age (years)	—									
SA (I)	−0.027	—								
CA (I)	0.043	0.058	—							
SC (I)	−0.097	−0.230	−0.081	—						
SA (D)	0.051	0.451 *	−0.044	−0.357 *	—					
CA (D)	0.076	−0.031	0.378	0.193	0.124	—				
SC (D)	−0.056	−0.284	0.038	0.548 *	−0.297	−0.061	—			
BW (kg)	0.307	0.120	0.172	−0.292	−0.168	−0.052	−0.190	—		
∑7 skinfolds	−0.116	−0.238	−0.062	0.090	−0.430 *	−0.002	0.052	0.340	—	
BF (%)	−0.141	−0.259	−0.023	0.135	−0.475 *	−0.038	0.097	0.257	0.959 **	—

Pearson’s correlation coefficient is shown, with 95% confidence intervals. * *p* < 0.05, ** *p* < 0.001; SA (I) = Somatic Anxiety Intensity; CA (I) = Cognitive Anxiety (Intensity); SC (I) = Self-confidence (Intensity); SA (D) = Somatic Anxiety (Directionality); CA (D) = Cognitive Anxiety (Directionality; SC (D) = Self-confidence (Directionality); BW = Body Weight; ∑ = summation; BF = Body fat.

## Data Availability

The data presented in this study are available on request from the corresponding author. The data are not publicly available due to being personal health information.
